# New Dye-Sensitized Solar Cells Obtained from Extracted Bracts of Bougainvillea Glabra and Spectabilis Betalain Pigments by Different Purification Processes

**DOI:** 10.3390/ijms12095565

**Published:** 2011-08-30

**Authors:** Angel Ramon Hernandez-Martinez, Miriam Estevez, Susana Vargas, Fracisco Quintanilla, Rogelio Rodriguez

**Affiliations:** 1 Centro de Física Aplicada y Tecnología Avanzada, Universidad Nacional Autónoma de México, Campus Juriquilla, Boulevard Juriquilla No. 3001, CP 76230, Juriquilla, Querétaro, Mexico; E-Mails: arhm@fata.unam.mx (A.R.H.-M.); miries@fata.unam.mx (M.E.); vmsu@servidor.unam.mx (S.V.); 2 Ciencias de la Salud, Universidad del Valle de México, Campus Querétaro, Boulevard Villas del Mesón No. 1000, CP 76230, Juriquilla, Querétaro, Mexico; E-Mail: franciscoqg_7@hotmail.com

**Keywords:** dye-sensitized solar cell, betalain, natural dyes, solar energy, dip coating

## Abstract

The performance of a new dye-sensitized solar cell (DSSC) based in a natural dye extracted from the Bougainvillea spectabilis’ bracts, is reported. The performance of this solar cell was compared with cells prepared using extract of the Bougainvillea glabra and mixture of both extracts; in both cases the pigments were betalains, obtained from Reddish-purple extract. These dyes were purified to different extents and used for the construction of solar cells that were electrically characterized. The materials were characterized using FTIR and UV-Vis. Solar cells were assembled using TiO_2_ thin film on indium tin oxide (ITO)-coated glass; a mesoporous film was sensitized with the Bougainvillea extracts. The obtained solar energy conversion efficiency was of 0.48% with a current density *J*_SC_ of 2.29 mA/cm^2^ using an irradiation of 100 mW/cm^2^ at 25 °C.

## 1. Introduction

Dye-sensitized solar cells (DSSCs) are the third generation of photovoltaic devices for the conversion of visible light in electric energy. These new types of solar cells are based on the photosensitization produced by the dyes on wide band-gap mesoporous metal oxide semiconductors; this sensitization is produced by the dye absorption of part of the visible light spectrum. One aspect of these DSSCs photocells that is particularly attractive, is the low cost of the solar energy conversion into electricity; this is possible mainly due to the use of inexpensive materials and the relative ease of the fabrication processes [1–3].

Recent studies have shown that metal oxides such as ZnO [4–6], SnO_2_ [4,7,8], Nb_2_O_5_ [4,9], but mainly TiO_2_, have been successfully used as photo-anode when a dye is absorbed in the interior of the porous layer [10]. The performance of DSSCs can be understood as a competition between two principal redox processes: electrons injection with rate constants of the order of picoseconds (10^−15^ to 10^−12^ s) and the regeneration of the oxidized dye with rate constants of the order of nanoseconds (10^−7^ to 10^−9^ s) [4]. The injected electrons are transported through the TiO_2_ film to a transparent electrode, while a redox-active electrolyte of I^−^/I_3_^−^ is used to reduce the dye cation charge and transport the resulting positive charge to a counter-electrode; however, before this, the photo-induced electron injection from the sensitizer dye to the TiO_2_ film conduction band, initiates the charge separation [10,11]. In this sense, the sensitized dye acts as the photo-driven electron pump of the device.

Several inorganic, organic and hybrid compounds have been investigated as sensitizer, including porphyrins [12], phtalocyanines [13,14], platinum complexes [15], fluorescent dyes [16], among others. Ru-based complexes sensitizers have been widely used because they have better efficiency and high durability. However these advantages are offset by their high expense and the tendency to undergo degradation in presence of water [17].

The use of natural pigments as sensitizing dye for the conversion of solar energy in electricity is very interesting because, on one hand they enhance the economical aspect and on the other, produce significant benefits from the environmental point of view [18,19]. Natural pigments extracted from fruits and vegetables [20–22], such as chlorophyll and anthocyanins, have been extensively investigated as DSSCs sensitizer.

**Figure 1:**
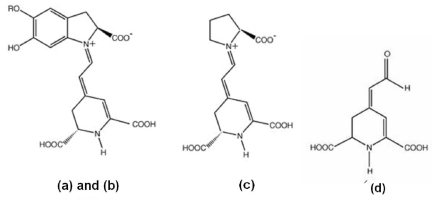
Schematic structures of betacyanin (**a**) Betanin R = β-D-glucose; (**b**) Betanidin R = H; (**c**) Indicaxanthin; (**d**) Betalamic Acid.

Two recent reports focused on the study of betalain pigments as DSSCs dyes sensitizer [17,20]. These pigments are present in flowers petals [17,20,23], fruits [17,18,24], leaves, stems and roots [20] of the Caryophyllales plants; they have high molar extinction coefficients in the visible region and pH-dependent redox properties [17,23,24]. The betalain pigments derived from betalamic acid are divided in two subgroups: the red betacyanins with maximum absorptivity at λ ≈ 535 nm and the yellow betaxanthins with maximum absorptivity at λ ≈ 480 nm. The schematic structures of betacyanin (red-purple): (a) betanin; (b) betanidin; (c) indicaxanthin (yellow-orange); and (d) betalamic acid, are reported in Figure 1 [17]. As shown in this figure, both dyes contain carboxylic groups that facilitate the link to the TiO_2_ surface. Betalain can be easily obtained from Bougainvillea bracts plants. The plants bracts are characterized by the presence of betacyanins [25] and betaxanthins [26]. These plants often grow in temperate climates having small flowers enclosed by large, brilliant red or purple bracts (modified leaves). However, there are relatively few reports about the use of betalain compounds as DSSCs sensitizer dyes, and most of them used beet [17] and Prickly pear fruit [20] as sources of these betalain compounds. Other sources of betalain as Pitaya (*Stenocereus thurberi*), Garambullo (*Myrtillocactus geometrizans*), Bougainvillea and Gooseberry, have been studied as food dye additives, however their performance has not been profoundly studied as cell sensitizers dyes. A recent study reports the performance of betalain extracts obtained from the Prickly pear fruit as a sensitizer which was compared with the Bougainvillea extract [20]. Except for this study, no more reports were found about the use of Bougainvillea as a source for these sensitizing dyes, although this offers more advantages over the Prickly pear fruit and the beet. The Genus Bougainvillea consist of about fourteen shrubby species indigenous of South America with inconspicuous flowers enclosed by showy bracts whose color ranges from white, yellow, orange, various shades of red to purple and violet [27].

**Figure 2:**
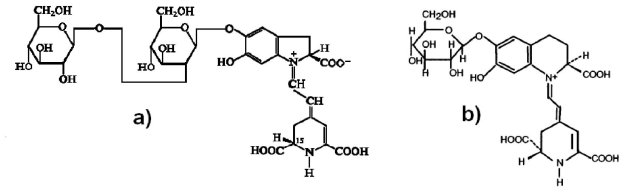
Chemical structures (**a**) bougainvillein-r-I (betanidin 5-*O*-β-sophorosides) and (**b**) betanin.

In a previous work [28] it has been reported, using paper electrophoresis and chromatography, the presence of two betacyanins in Bougainvillea spectabilis and four in Bougainvillea glabra. Later, by the use of chromatography on polyamide powder and high-voltage electrophoresis, some authors [29] found eleven violet-red pigments (bougainvillein-v’s) in Bougainvillea glabra with lambda max from 522 to 551 nm, the main difference between the pigments found in the Bougainvillea and another source of betalain pigments is the saccharide type present in the betanidin; Figure 2 shows the molecular structure of (a) bougainvillein-r-I (betanidin 5-*O*-β-sophorosides) [23] and (b) betanin [24]. The two main varieties of Bougainvillea, Bougainvillea glabra (*Nyctainaceae*) and Bougainvillea spectabilis, are easily available and low cost ornamental plants. In some cases they are used in traditional medicine [25], but usually they do not have commercial or nutritious use; so there is no conflict for their use in energy production. Additionally, they flourish all year.

The extract purification process might increase the manufacturing costs of the cells. In consequence, one of the objectives of this work is addressed to analyze the performance of specific solar cells with different purification steps in order to reduce costs. Then, in order to find the dye with a higher performance, our work reports the results obtained for DSSCs assembled using raw extracts of Bougainvillea spectabilis and glabra.

## 2. Experimental Section

### 2.1. Preparation of Dye-Sensitizer Solutions

Fresh Bougainvillea glabra and spectabilis flowers were harvested in central Mexico (Queretaro). Violet and red bracts were used from both species. All flowers’ juices were prepared by grinding 12 g of dried flowers in 500 mL of acid water with pH = 5.7; these extracts were filtered and centrifuged separately to remove any solid residue and stabilized at pH = 5.7 by the addition of an aqueous solution 1 N of HCl. Two different extracts from red Bougainvillea glabra (RBG) were used: the first one was taken from the half of the extract and purified using an Amberlited packed column and ammonium acetate (CH_3_COONH_4_) 1 M in acid water as the eluting solvent to leave only the yellow and red betalain fractions; the second half was used as prepared to determine the efficiency in the sensitization effect that can be reached with minimal chemical procedure and whether the presence of other compounds, in addition to the dye itself, do not interfere with the performed of the dye. The rest of the extracts: Violet Bougainvillea glabra (VBG), Red Bougainvillea spectabilis (RBS), and Violet Bougainvillea spectabilis (VBS), were used without further purification. The dye solutions in acid conditions (pH = 5.7) are stable with a half-time deactivation of more than 12 months when they are properly stored, protected from direct sunlight and refrigerated at about 4 °C [30]. The betaxanthin content was determined using UV-Vis spectroscopy.

### 2.2. Electrodes Preparation

The conductive glass plates were indium tin oxide (ITO)-coated glass slides (In_2_O_3_:SnO_2_) with a sheet resistance of 30–60 Ω/cm^2^, 84% transmittance nominal at 550 nm, dimensions (L,W,D) of 25 × 25 × 1.1 mm. Titanium oxide (TiO_2_) nanopowder (mesh 320) (Aldrich) and the solvents, ethanol and acetonitrile (Aldrich), were analytic grade; they were used as received. ITO substrates were ultrasonically cleaned in an ethanol-water mixture for 30 min and then heated at 450 °C during 30 min prior to film deposition. The photo-anodes were prepared by depositing two TiO_2_ films on the cleaned ITO glass: for the first one the ITO glass was immersed in a sol-gel solution containing a mixture of titanium isopropoxide, water and isopropanol at concentration 2:1:25 vol.; the immersion was at constant speed (1.5 cm/min) [31]; the coated glass was heated at 450 °C during 30 min producing a densified TiO_2_ thin film; this isolate the dye-activated porous TiO_2_ layer from the conductor glass. Subsequently two edges of the ITO glass plate were covered with adhesive tape (Scotch 3M) to control the thickness of the film; finally a TiO_2_ paste was spread uniformly on the substrate by sliding a glass rod along the tape spacer. The TiO_2_ paste was prepared by mixing 3.0 g of TiO_2_ nano-powder, 10 mL of nitric acid 0.1 N and 4 mL of polyethylene glycol; this suspension was stirred in a closed glass container for 24 h to obtain a smooth paste with the appropriate viscosity. The film was heated at 450 °C for 60 min resulting in a mesoporous film with a thickness of around 8–10 μm and opaque. The TiO_2_ photo-anodes were first soaked for 12 h in HCl and then immersed in the natural dye solutions for one night at room temperature, according to published procedures [17]. Later, the photo-anodes were rinsed with distilled water and ethanol and dried. Carbon coated counter electrodes were prepared following a procedure reported elsewhere [32].

### 2.3. DSSC Assembling

An electrolyte solution was prepared as reported elsewhere [17]: 0.1 M of I_2_ was mixed with 0.05 M of LiI and 0.05 M of 3-methoxypropionitrile in 50 mL of acetonitrile (C_2_H_3_N) and stirring for 60 min. This electrolyte solution was poured in the mesoporous TiO_2_ film which was previously prepared using paraffin-film as framework to seal the cells to prevent evaporation of the liquids. The counter electrode was pressed against the impregnated anode and clamped firmly in a sandwich configuration. No leaks (solvent evaporation) were detected.

### 2.4. Characterization

The topology of the TiO_2_ films was obtained using a scanning electron microscope (SEM) Jeol JSM-6060LV operated at 20 kV in secondary electron mode with different magnifications; the samples were covered with a gold film. The particle size and the particle size distribution of the commercial TiO_2_ particles were determined using a light scattering apparatus Brookhaven Instruments model BI200SM, equipped with a high speed digital correlator PCI-BI9000AT, a solid-state photon detector and a He-Ne laser of 35 mW Melles Griot 9167EB-1 as a light source. The crystalline structure of the TiO_2_ thin film was determined using a diffractometer Rigaku model Miniflex+ equipped with a radiation source of 1.54 Å and the angle 2θ was varied from 5° to 80° at a scan of 2°/min. Absorption spectra were obtained using a UV-Vis spectrometer Genesys 2PC. The DSSCs were illuminated using a 75 W Halogen lamp with an incident power of about 100 mW/cm^2^ in an illumination area of 0.16 cm^2^; UV and IR filter glasses were used in front of the sample. Photocurrent and photovoltage were measured using a Keithley 2400 source meter and the incident light power was determined using a Powermeter Thor Labs S130A (0–300 mw) and a Spectrometer Ocean Optics HR 400.

## 3. Results and Discussion

### 3.1. Absorption Spectra

**Figure 3:**
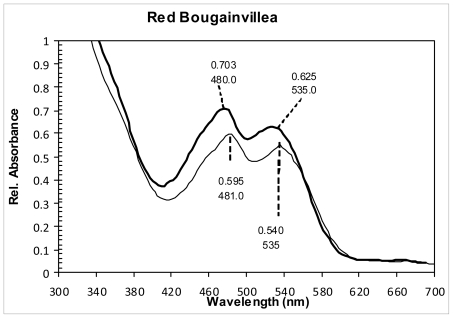
Absorption spectra of red Bougainvillea spectabilis (RBS) extract (thick line) and red Bougainvillea glabra (RBG) extract (thin line), both diluted in water at pH = 5.7.

Figure 3 shows the absorption spectra of RBS and RBG extracts, both diluted in water at pH = 5.7. In both cases two peaks were found: the first one around 480 nm (480 nm for RBS and 481 nm for RBG) can be associated to the presence of indicaxanthin, while the second one at 535 nm is attributable to the betanin or the bougainvillein-r.

The indicaxanthin concentration in μM was estimated from the absorbance peaks at 481 and 535 nm using the expression [24,33]:

$$(\text{Indicaxanthin})=23.8A_{482}-7.7A_{536}$$

**Figure 4:**
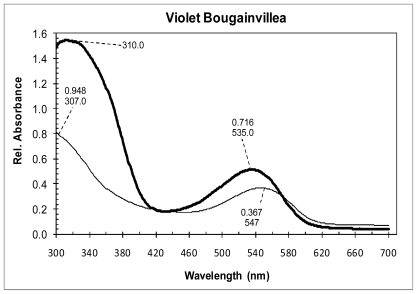
Absorption spectra of violet Bougainvillea spectabilis (VBS) extract (thick line) and violet Bougainvillea glabra (VBG) extract (thin line), both diluted in water at pH = 5.7.

The extracted and diluted VBS and VBG solutions were analyzed with the spectrophotometer at wavelengths between 260 and 700 nm. The absorption spectrum showed two major absorption peaks at 310 and 535 nm for VBS extract and at 307 and 547 nm for VBG extract (Figure 4). Absorbance peaks at 300 and 535 nm are characteristic absorptions for red violet betalain group, betacyanin [34], for bougainvillein-r the absorbance peaks are 280, 320 and 541 nm [23].

The substrates were immersed for 12 h in a 1 M HCl solution to facilitate the absorption of betacyanins on betaxanthins; it is known [17] that the betacyanins absorption is inhibited by the presence of indicaxina. A dip in HCl results in the protonation of the betalainic carboxyl group from its anionic form, promoting its absorption in the TiO_2_ film.

### 3.2. Structure and Surface Characteristic

**Figure 5:**
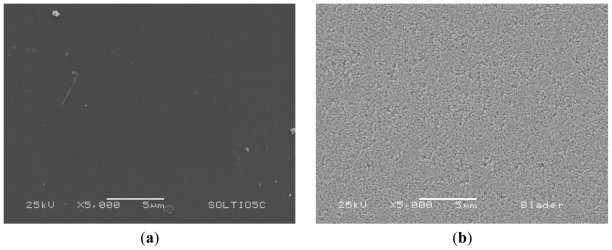
SEM images (**a**) TiO_2_ compact layer overlying conductive glass, and (**b**) second nanoporous TiO_2_ layer formed by screen-printing technique.

The particle size and the particle size distribution of the commercial TiO_2_ particles were determined by dynamic light scattering (DLS) resulting of (285 ± 15) nm. The TiO_2_ surface morphology is shown in Figure 5a,b. Figure 5a shows a compact (no pores) structure of the first thin layer of TiO_2_ obtained by immersing the ITO plates in a titanium alkoxide sol-gel precursor. This thin film acts as an insulator between the ITO layer and the dye-activated porous TiO_2_ layer. Some authors [20] have proved the usefulness of the compact layer to achieve a higher efficiency in the performance of the DSSC. On the other side, Figure 5b shows the surface of the thick TiO_2_ coating obtained by the screen-printing technique; here it is possible to see a homogeneous mesoporous surface formed by the TiO_2_ nanoparticles. The nanoporous structure is useful because it has a high effective surface area where the molecules of the Bougainvillea extracts can be linked.

### 3.3. Photoelectrochemistry

Five different Bougainvillea extracts were investigated. The first four correspond to extracts obtained from: RBG, VBG, RBS and VBS. They were centrifuged, filtered and used fresh without further purification, as explained above. The fifth one was Red Bougainvillea Spectabilis extract freshly centrifuged and purified by a packed column to remove the bulk of free sucrose compounds; this extract was named PRBS.

Table 1 summarize the results for the five extracts; in all cases low currents were obtained, but comparatively higher compared to those obtained by other authors [17]; this probably partially originated as a result of using ITO-glass with sheet resistance of 30–60 Ohm cm^−2^ instead of Fluorine doped tin oxide (FTO) glass with sheet resistance of 10–15 Ohm cm^−2^ as reported elsewhere [20]. In this table the short-circuit photocurrent density *J*_SC_, the current density at maximum power *J*_MP_, the open-circuit voltage *V*_OC_, the maximum power Voltage *V*_MP_, the maximum power *P*_M_, the theoretical power *P*_T_, the fill factor *FF*, and the energy conversion efficiency η, are reported.

**Figure 6:**
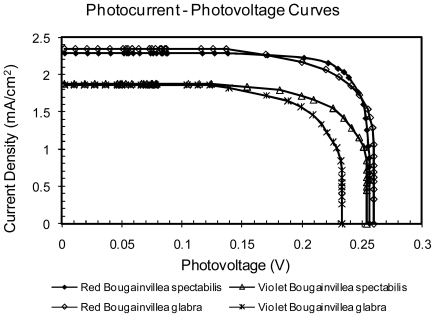
Photocurrent-photovoltage curves for a Bougainvillea extract sensitized solar cell.

**Figure 7:**
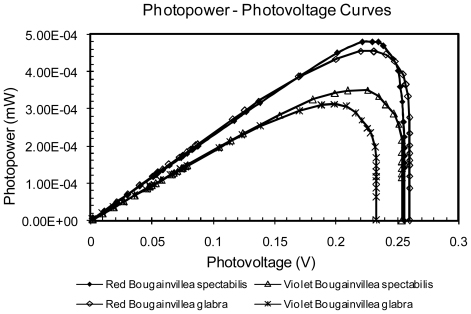
Power-photovoltage curves for a Bougainvillea extract sensitized solar cell.

It was found that regardless of the species of Bougainvillea used, the extract with the best performance parameters comes from the red bracts (RBG and RBS) as shown in Figure 6. This is because these extracts have both betaxanthin and betacyanins; each with absorptions at different wavelengths, helping the cell to capture photons of two different energies. Figure 7 shows the power curves obtained in terms of voltage; it is possible to observe again the improved sensitivity provided by red bracts compared to the violet ones.

Bougainvillea extracts displayed interesting photoelectrochemical performance parameters: *J*_SC_ close to 2.3 mA/cm^2^; *V*_OC_ near to 0.26 V; an excellent fill factor of 0.74; and η in the neighborhood of 0.49% using an electrolyte composed of 0.1 M I_2_/0.05 M LiI/0.05 M 3-methoxypropionitrile in acetonitrile (C_2_H_3_N). The application of a compact TiO_2_ under-layer (*i.e.*, overcrowding layer) was necessary for enhancing the cell performance, as compared with previously reported yields [17,20] (Table 1). The filling factor found in this research is promising because, to our knowledge, it is among the highest so far reported with raw natural dyes.

**Figure 8:**
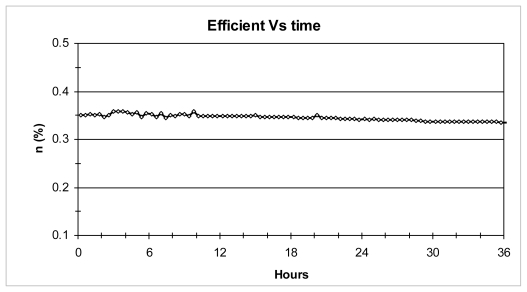
Time dependence of the efficiency for Bougainvillea extract sensitized solar cell.

Additionally, a test on the stability of these natural dyes was carried out by monitoring some indicative parameters, such as *J*_SC_ and η, under continuous sun illumination (100 mW/cm^2^ and air mass 1.5) in a hermetically sealed solar cell with an electrolyte solution (0.1 M of I_2_, 0.05 M of LiI, 0.05 M of 3-methoxypropionitrile in acetonitrile) and without any cooling system during 36 h; no significant changes were observed in this time as reported in Figure 8.

## 4. Conclusions

The Bougainvillea spectabilis and glabra extracts were studied as natural dyes for DSSCs. The results are consistent with those reported previously for Bougainvillea; however a higher *FF* was obtained due to the presence of the insulating TiO_2_ layer; additionally an electrolyte with less vapor pressure increases the lifetime of the solar cell. One important finding was that the mixture of betaxanthin and betacyanin produces a better conversion than betacyanin alone: the absorption at different wavelengths of betaxanthin and betacyanin increases the absorption of photons of different energies. A thorough study of the ratio betaxanthin-betacyanin in order to obtain the highest efficiency in these dye-sensitized solar cells is required. It was also found that the use of a highly purified extract does not represent a significant increment in cell efficiency, but it is possible that the presence of other compounds than the dye itself, e.g., sucrose, can affect the lifetime of the solar cell. As each extract from a particular source will have a different compound combination or different betalain compounds, it is important to characterize the extract from different sources in order to find the best efficiency at the lowest cost. In this particular case, a simple purification process to separate sucrose free compounds produced a device with the lowest cost and highest efficiency.

## Figures and Tables

**Table 1:** Electrical characterization of all prepared cells.

Dye Source *	*J*_sc_ (mA/cm^2^)	*J*_MP_ (mA/cm^2^)	*V*_OC_ (V)	*V*_MP_ (V)	*P*_M_ (μW)	*P*_T_ (μW)	*FF*	η (%)
RBG	2.344	1.966	0.26	0.231	0.45	0.60	0.74	0.45
VBG	1.860	1.568	0.23	0.199	0.31	0.43	0.71	0.31
RBS	2.291	2.083	0.28	0.229	0.48	0.63	0.76	0.48
VBS	1.881	1.552	0.25	0.226	0.35	0.48	0.73	0.35
PRBS	2.327	2.12	0.26	0.228	0.48	0.61	0.79	0.49

* Red Bougainvillea glabra (RBS), Violet Bougainvillea glabra (VBG), Red Bougainvillea spectabilis (RBS), Violet Bougainvillea spectabilis (VBS), and Purified Red Bougainvillea glabra (PRBS).
